# Supplementary material for: ENPP1 variants in patients with GACI and PXE expand the clinical and genetic heterogeneity of heritable disorders of ectopic calcification
Source: PLoS Genet. 2022 Apr 28;18(4):e1010192. doi: 10.1371/journal.pgen.1010192 (PMC9089899; doi:10.1371/journal.pgen.1010192)
Supplement: S1 Text — (DOCX) [file pgen.1010192.s001.docx]

**S1 Text**

**Clinical features and biochemical findings of GACI patients**

Family #1 was a consanguineous Pakistani family with one affected male, patient #1 (Fig 1a). The parents were first cousins with three other healthy children. Patient #1 was diagnosed *in utero* with GACI by prenatal ultrasound, showing moderate pericardial effusion and calcification of aorta and superior mesenteric artery (Fig 2a1). A fetal magnetic resonance imaging of the brain was unremarkable. He was born at 38 weeks of gestation by elective Caesarian section with respiratory distress and stayed in the neonatal intensive care unit until 13 days of age. At three days after birth, chest X-ray revealed calcification of pulmonary artery, descending and abdominal aorta (Fig 2a2). His neonatal blood work showed normal mineral homeostasis except elevated FGF23 (S1 Table). He began treatment with oral etidronate (6.5 mg four times per day) and cholecalciferol (400 IU per day) at six days of age. The family lost insurance and was not able to do more follow-ups. He died at five months of age due to sudden cardiac death.

The consanguineous family #2 from Pakistan had three children; two of them were affected with GACI (Fig 1b). Patient #2 was born by an emergent Caesarian section at 33 weeks of gestation due to worsening hydrops and cardiac profile score, and she died a few hours after birth. An autopsy was suggestive of GACI. Because of the family history of GACI, during the third pregnancy the mother took etidronate four times per day until ten days before delivery. Patient #3 with GACI was born clinically stable, and he received 12.5 g/m^2^ sodium thiosulfate five times per week from nine days of age for one year. Repeated computed tomography angiography (CTA) and ultrasound from three days to three months of age revealed calcification of the abdominal aorta, iliac, and renal arteries (Fig 2b). There is also abnormal calcification of the left proximal femoral epiphysis. CTA at nine months of age showed new soft tissue calcification in the right subscapular region and the bilateral heads of the clavicle. There was mild stenosis of the aorta proximal to its bifurcation. He was diagnosed with hypophosphatemic rickets at 13 months of age for which he was treated with 45 mg/kg phosphorus, four times a day, and 40 mcg/kg/day calcitriol. CTA at 16 months of age showed no new or residual arterial calcification with stable clavicles calcification and reduced calcification in the right subscapular region. His blood work at one day of life showed elevated ALP but Ca, Pi and PTH were all within normal limits (S1 Table). Serum Pi level was consistently low starting at one month of age over the first three years of life, while ALP activity was consistently high (S1 Table). He is now three years of age.

Family #3 was a Caucasian family in which three of six children presented with features of GACI (Fig 1c). Patient #4 was healthy at birth but developed respiratory distress and died at 49 days of age due to heart failure. The diagnosis of GACI was confirmed by autopsy showing generalized arterial calcification as well as myocardial infarction and cardiomegaly. Patient #5 died of myocardial ischemia at 38 days of life. Prenatal ultrasound of fetus #6 at 28 weeks of gestation showed extensive calcification involving the common iliac arteries and abdominal aorta with severe stenosis (Fig 2c1). During pregnancy, the mother began on 2 mg/kg of etidronate per day at 31 weeks of gestation. Weekly fetal ultrasound showed no further progression of fetal arterial calcification during the rest of pregnancy. Patient #6 was born stable at 39 weeks of gestation. After birth, repeated CTA and echocardiogram showed reduced arterial calcification (Fig 2c2), but severe arterial stenosis was still noted (Fig 2c3). Her serum FGF23 level was elevated (S1 Table). At 21 days of age, she was given 12.5 g/m^2^ daily sodium thiosulfate infusions five times per week for her first year of life. She was diagnosed with hypophosphatemic rickets at 24 months of age (Fig 2c4). She started to receive calcitriol and phosphorus treatment, with improved serum Pi, although still below the normal range (S1 Table). Repeated follow-up CTA up to three years of age showed only residual arterial calcification, but stenosis was still present. There was no evidence of new areas of arterial calcification. She is now eight years of age and has attained all developmental milestones with normal cardiac function without evidence of neurological impairment or hypertension.

Family #4 is a Pakistani family with three affected children with GACI (Fig 1d). Patient #7 was born with an absent left hand. He was seen at two years of age due to bowed legs and anklets, characteristics of rickets. Blood work showed low Pi, high ALP, and FGF23 (S1 Table). Since age three, he has been treated with phosphorus and alfacalcidol; however, his serum ALP remained high, and serum Pi remained low (S1 Table). Repeated ultrasound showed no calcification of systemic arteries but did show stenosis of the left carotid artery. At age four, he developed a respiratory tract infection and died suddenly. An autopsy showed bronchopneumonia, ischemia and myocardial fibrosis, without calcification in any major blood vessels. Patient #8 had multiple prenatal ultrasounds, and she was diagnosed with GACI by the presence of arterial calcification at 30 weeks of gestation. The mother took etidronate at 32 weeks of gestation until patient #8 was born by a Caesarian section at 35 weeks of gestation. Postnatal X-ray and CTA of patient #8 revealed calcification of the axillary arteries, the scapular region of the shoulders (Fig 2d1), and the abdominal aorta extending into bilateral iliac arteries (Fig 2d2). She is currently two years of age. Patient #9 was diagnosed with GACI at two weeks of age by widespread arterial calcification accompanied by hypertension. Despite receiving treatment of sodium thiosulfate and etidronate starting at three weeks of age, he passed away at three months of age.

Family #5 had one affected Chinese male, patient #10 (Fig 1e), who was born by elective Caesarian section due to fetal distress at 36 weeks of gestation. An echocardiogram at one day of life showed an enlarged left ventricle. He was administered digoxin for three months and metoprolol for six years. While the cardiac hypertrophy was largely under control, he regularly complained of intermittent pain in both lower extremities. Because of hypertension and a fall at age 6.5 years, he was admitted to the hospital. Dynamic electrocardiography showed sinus arrhythmia, sinus bradycardia and prolonged PR interval. CTA showed stenosis of the abdominal aorta and bilateral renal arteries without evidence of calcification (Fig 2e1). Upon physical examination, he had bowed legs, and the radiograph demonstrated features of rickets (Fig 2e2). He was subsequently diagnosed with GACI. His blood work showed low Pi and high ALP (S1 Table). He took oral phosphorus 3 ml every four hours together with alfacalcidol soft capsule 0.25 µg per day, and the serum Pi level was marginally improved but still below the normal range (S1 Table). Due to continued hypertension, he underwent two balloon dilatation surgeries. He is currently eight years of age, and his hypertension is currently under control.
