# Supplementary material for: ENPP1 variants in patients with GACI and PXE expand the clinical and genetic heterogeneity of heritable disorders of ectopic calcification
Source: PLoS Genet. 2022 Apr 28;18(4):e1010192. doi: 10.1371/journal.pgen.1010192 (PMC9089899; doi:10.1371/journal.pgen.1010192)
Supplement: S1 Table — (DOCX) [file pgen.1010192.s002.docx]

**S1 Table. Biochemical findings of patients with *ENPP1* variants**

| **Family ID** | **Patient ID** | **Age at analysis** | **Ca (mg/dL)**  **(8.8-11.3) 0-0.25 yrs**  **(9.4-10.8) 1-5 yrs**  **(9.4-10.3) 6-12 yrs**  **(8.8-10.2) 13-20 yrs** | **Pi (mg/dL)**  **(4.8-7.4) 0-0.25 yrs**  **(4.5-6.5) 1-5 yrs**  **(3.6-5.8) 6-12 yrs**  **(2.3-4.5) 13-20 yrs** | **ALP (U/L)**  **(100-350) 1-5 yrs**  **(60-450) 6-12 yrs**  **(40-180) 13-20 yrs** | **FGF23**  **C-terminal and whole molecule <270 RU/mL** | **PTH**  **(12-65 pg/mL)** | **25(OH)D3**  **(20-80 ng/mL)** |
| --- | --- | --- | --- | --- | --- | --- | --- | --- |
| Family #1 | Patient #1 | 1-12 day | 9.6 | 5.8 | 205 | 1,075↑ | 32.5 | 52.4 |
| Family #2 | Patient #3 | 1 day | 8.8 | 5.4 | 930↑ |  | 27.0 | 16.6↓ |
|  |  | 1-13 mo | 9.8 | 2.9↓ | 378↑ | 240 | 16.0 | 31.6 |
|  |  | 13-36 mo | 9.6 | 3.2↓ | 591↑ |  | 50.0 | 27.0 |
| Family #3 | Patient #6 | 1 day-8 mo | 9.7 | 5.0 | 302 | 321↑ | 27.1 | 58.0 |
|  |  | 1-2.5 yr | 9.7 | 3.2↓ | 443↑ |  | 76.5 | 24.4 |
|  |  | 3-4 yr | 9.2 | 4.0↓ | 278 |  | 23.6 | 30.5 |
| Family #4 | Patient #7 | prior to 2 yr | 9.5 | 3.5↓ | 1,392↑ |  | normal |  |
|  |  | 2 yr | 9.9 | 3.9↓ | 1,781↑ | 278↑ | normal | 19.2↓ |
|  |  | 3.5 yr | 9.9 | 2.8↓ | 1,816↑ | 112 | normal | 16.4↓ |
| Family #5 | Patient #10 | 6.5 yr | 9.8 | 2.3↓ | 471↑ |  | 19.6 | 28.6 |
|  |  | 7-8 yr | 8.9 | 2.9↓ | 442 |  | 59.9 |  |
| Family #6 | Patient #11 | 57 yr | 9.1 |  | 98 |  |  |  |
| Family #7 | Patient #12 | 14-21 yr | 9.5 | 3.6 | 141 |  |  | 63.4 |
