# Supplementary material for: ENPP1 variants in patients with GACI and PXE expand the clinical and genetic heterogeneity of heritable disorders of ectopic calcification
Source: PLoS Genet. 2022 Apr 28;18(4):e1010192. doi: 10.1371/journal.pgen.1010192 (PMC9089899; doi:10.1371/journal.pgen.1010192)
Supplement: Attachment — Submitted filename: rebuttal letter R1.pdf [file pgen.1010192.s003.pdf]

Jefferson
Philadelphia University + Thomas Jefferson University
HOME OF SIDNEY KIMMEL MEDICAL COLLEGE

Department of Dermatology and Cutaneous Biology
233 South 10th Street
Bluemle Life Sciences Building
Suite 450
Philadelphia, PA 19107
T 215-503-5785
F 215-503-5788

3/9/2022

Melissa Wasserstein, M.D.
Associate Editor
PLOS GENETICS

Gregory S. Barsh, M.D., Ph.D.
Editor-in-Chief
PLOS GENETICS

RE: PGENETICS-D-21-01563R1
"*ENPP1* variants in patients with GACI and PXE expand the clinical and genetic heterogeneity of heritable disorders of ectopic calcification" (Ralph *et al*.)

Dear Drs. Wasserstein and Barsh,

Thank you for your email of February 24, 2022, containing the Editors' and Reviewers' comments on our submission referenced above. We noted that "*The reviewers appreciated the attention to an important topic but identified some concerns that we ask you address in a revised manuscript*". We would like to thank the Editors and Reviewers for their constructive suggestions.

We have carefully examined the comments, and our responses to the issues raised are provided below. Enclosed please find a revised manuscript in which all these changes are tracked by red.

Editors:

- *While revising your submission, please upload your figure files to the Preflight Analysis and Conversion Engine (PACE) digital diagnostic tool.*

Reply: As requested, we have uploaded all figures to PACE.

- *Please be aware that our data availability policy requires that all numerical data underlying graphs or summary statistics are included with the submission, and you will need to provide this upon resubmission if not already present.*

Reply: In the revision, we have uploaded all raw numerical data and summary statistics as a supplementary material.

- *To enhance the reproducibility of your results, we recommend that you deposit your laboratory protocols in protocols.io, where a protocol can be assigned its own identifier (DOI) such that it can be cited independently in the future.*

Reply: This does not apply to our submission as none of the protocols used in our study are new. They are well established and used worldwide.

- *Please review your reference list to ensure that it is complete and correct.*

Reply: We confirm that all references are complete and correct.

Reviewer: 1

- *The authors reports the clinical, laboratory, and molecular evaluations of ten GACI and two PXE patients from five and two unrelated families registered in GACI Global and PXE International databases, respectively. The authors conclude that the phenotypic spectrum of ENPP1-deficiency is much broader than was previously anticipated. It is known that GACI and PXE are complex disease, and the genes involved have many modifiers, e.g. doi: 10.3389/fcell.2021.612581. The authors further state that the correlation of plasma PPi and severity of GACI and PXE may not hold. The content of the paper could be greatly increased if the authors discuss alternative disease mechanisms beside the long-held association of extracellular PPi and calcification inhibition. Circulating PPi may be a poor proxy of the local PPi concentrations, which may actually determine the cellular calcification milieu. Also, cleavage of each ATP releases AMP along with PPi. What ist known about the role of AMP signalling in these diseases? Adenine, and specific ribonucleosides that disrupt pyrimidine synthesis may regulate the severity of GACI and PXE by affecting cell survival. (Li et al https://doi.org/10.1172/JCI149711). Please discuss.*

Reply: We have now elaborated in more detail in discussion towards the phenotypic heterogeneity and the lack of correlation between plasma PPi concentrations and severity of ectopic calcification (Discussion, page 15): "Several potential mechanisms may explain the phenotypic heterogeneity and the poor correlation between plasma PPi concentrations and disease severity. First, environmental factors and genetic modifiers may influence the disease severity of ectopic calcification (2, 32). Secondly, although we cannot currently measure extracellular PPi levels in tissues, circulating PPi may be a poor proxy of the local PPi concentrations which may be more important in preventing tissue calcification. Thirdly, in addition to PPi, ENPP1-mediated hydrolysis of ATP also produces adenosine monophosphate. The pathophysiologic role of adenosine monophosphate in the disease process of GACI was recently reported (9). Furthermore, the potential dysregulation of extracellular nucleotide metabolism, for example, ENPP1-mediated disruption of pyrimidine synthesis known to regulate tissue repair, may play a role in ectopic tissue calcification (33)." We also included the two publications suggested by the reviewer as new citations, #32 and #33.

- *li 151 "The Family #7 had one adopted 27-year-old male, patient #12, of Caucasian and African American descent (Fig. 1g). He also seeks support from PXE International." The fact that an unrelated adopted child acquired similar affection to me suggests the contribution of environmental or nutritional factors. Is this possible or was the child adopted BECAUSE it was affected? Please explain.*

Reply: Patient #12 was adopted at birth as an apparently healthy infant. His adopted parents, who are clinically healthy, registered him in PXE International when he was 12 years of age. We have now modified the text as follows (Page 7): "The proband in Family #7, a 27-year-old male, patient #12 of Caucasian and African American descent, was adopted at birth (Fig. 1g). He is a member of PXE International when he was 12. The adopted parents are clinically healthy."

- *li 272 "elevated serum FGF23 levels in several GACI patients in the current study support this hypothesis" FGF-23 is a phosphatonin. Elevated levels may suggest phosphate and calciprotein particle toxicity with consequences for cell ageing and cell death (https://doi.org/10.1016/j.kint.2019.10.019 and papers cited therein). Please discuss.*

Reply: We have now modified the discussion as follows (Discussion, page 13): "It was suggested that ARHR2 is FGF23-mediated (3), and the elevated serum FGF23 levels in several GACI patients in the current study support this hypothesis. Elevated FGF23 levels may also be a response of cells to circulating calciprotein particles, which are associated with vascular calcification (25)." We also included the publication suggested by the reviewer as a new citation, #25.

Reviewer: 2

Minor comments to the work are as follows:

1. *For readers less familiar with this pathway, please provide a graphical representation for the pathways of interest in the introduction highlighting ABCC6, ENPP1, Pyrophosphate, ATP, etc.*

Reply: The pathways of PPi metabolism have been published in several review articles. We just had an update on the pathways in a review article published in *Am J Pathol*: Ralph et al. Inorganic pyrophosphate deficiency syndromes and potential treatments for pathologic tissue calcification. Am J Pathol. 2022 Feb 16:S0002-9440(22)00049-9. doi: 10.1016/j.ajpath.2022.01.012. Online ahead of print. PMID: 35182493. This most up-to-date review article is now included as a new citation, #1, in the revised manuscript.

2. *In the introduction both GACI1 and GACI are utilized- please keep consistent.*

Reply: We have now utilized "GACI" throughout the manuscript.

3. *On line 170 it indicates that patients 7, 8, and 9 are siblings. From the diagram in figure 1, is patient 9 a sibling or cousin? Please correct.*

Reply: The reviewer is correct: the patient #9 is a cousin. It is now corrected in the text.

4. *An additional paragraph in the results section connection PPi levels to phenotype is warranted. This is a main idea of the abstract and could be better discussed in the results, along side the PPi level measures. Are Phenodex type of values available for GACI patients?*

Reply: Phenodex scores are applicable to PXE patients only because PXE is a late-onset and systemic ectopic calcification disorder. The predominant phenotype in GACI patients is vascular calcification, occurring either *in utero* or in early infancy. Despite the clinically distinct phenotypes between typical PXE and GACI, there is no strict correlation between circulating PPi levels and disease severity. We have now discussed these findings in more detail in Discussion (page 15): "Several potential mechanisms may explain the phenotypic heterogeneity and the poor correlation between plasma PPi concentrations and disease severity. First, environmental factors and genetic modifiers may influence the disease severity of ectopic calcification (2, 32). Secondly, although we cannot currently measure extracellular PPi levels in tissues which may be more important in preventing tissue calcification, circulating PPi may be a poor proxy of the local PPi concentrations. Thirdly, in addition to PPi, ENPP1-mediated hydrolysis of ATP also produces adenosine monophosphate. The pathophysiologic role of adenosine monophosphate in the disease process of GACI was recently reported (9). Furthermore, the potential dysregulation of extracellular nucleotide metabolism, for example, ENPP1-mediated disruption of pyrimidine synthesis known to regulate tissue repair, may play a role in ectopic tissue calcification (33)."

5. *Please change blue arrows in Figure 2 to another color- maybe yellow, to increase visibility.*

Reply: We have now changed the blue arrows to yellow arrows.

6. *Please provide scale bars throughout Figure 2.*

Reply: The original clinical images from our clinical collaborators do not have scale bars. As we intended to highlight clinical findings, the lack of the absolute scale of the clinical pictures does not interfere with the comprehension of the clinical features. Our co-author, Dr. Levine, confirmed that these images do not carry scale bars.

7. *At the end of figure 2 legend, it denotes d, dead; a, alive – where is this denoted in the figure? All patients included alive, correct?*

Reply: We apologize for the confusion. The denotes of "*d, dead; a, alive*" apply to Figure 1 instead of Figure 2. We have now moved them to the legend of Figure 1.

8. *Please provide scale bars for 3d.*

Reply: We did provide the scale bars for images in Fig. 3d. We have now made the scale bars thicker and brighter and uploaded a new Figure 3 in our revision to increase readability.

9. *Please indicate how many biological or technical replicated were performed in Figure 3.*

Reply: The details are as follows:

Fig. 3a and 3b – Sanger sequencing: We did bidirectional sequencing for each variant.

Fig. 3c – ENPP1 Western blot: Three independent transfection experiments were performed. One technical replicate per transfection. The representative images are shown here.

Fig. 3d – ENPP1 IF staining: Three independent transfection experiments were performed. One technical replicate per transfection. The representative images are shown here.

Fig. 3e – ENPP1 enzyme activity: Three independent transfection experiments were performed. Three technical replicates for each transfection.

Fig. 3f – PPi quantification: Three independent transfection experiments were performed. One technical replicate for each transfection.

We have now provided this information in the legend to Figure 3. We also uploaded all raw numerical data and summary statistics reported in Fig. 3e and Fig. 3f as a supplementary material.

10. *Please expand in the methods the concentration of antibody used for both western and fluorescent cell-based analysis. 1:100? 1:1000? Additional details to help other reproduce such work is needed.*

Reply: We have now provided this information in the Materials and Methods section.

We believe that we have appropriately addressed the Editors' and Reviewers' concerns, and we would appreciate you reconsidering our revised manuscript for publication in *Plos Genetics*.

Thank you for your consideration. Looking forward to hearing from you soon.

Sincerely yours,

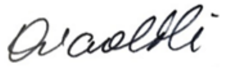

Qiaoli Li, Ph.D.
Professor
Department of Dermatology and Cutaneous Biology
